# Impact of Reset Pulse Width on Gradual Conductance Programming in Al_2_O_3_/TiO_x_-Based RRAM

**DOI:** 10.3390/mi16060718

**Published:** 2025-06-17

**Authors:** Hyeonseong Lim, Wonbo Shim, Tae-Hyeon Kim

**Affiliations:** 1 Department of Semiconductor Engineering, Seoul National University of Science and Technology, Seoul 01811, Republic of Korea; lhs12057@seoultech.ac.kr; 2 Department of Electrical and Information Engineering, Seoul National University of Science and Technology, Seoul 01811, Republic of Korea; wbshim@seoultech.ac.kr

**Keywords:** resistive random access memory (RRAM), resistive switching, incremental step pulse programming (ISPP)

## Abstract

This work investigates the impact of reset pulse width on multilevel conductance programming in Al_2_O_3_/TiO_x_-based resistive random access memory. A 32 × 32 cross-point array of Ti (12 nm)/Pt (62 nm)/Al_2_O_3_ (3 nm)/TiO_x_ (32 nm)/Ti (14 nm)/Pt (60 nm) devices (2.5 µm × 2.5 µm active area) was fabricated via e-beam evaporation, atomic layer deposition, and reactive sputtering. Following an initial forming step and a stabilization phase of five DC reset–set cycles, devices were programmed using an incremental step pulse programming (ISPP) scheme. Reset pulses of fixed amplitude were applied with widths of 100 µs, 10 µs, 1 µs, and 100 ns, and the programming sequence was terminated when the read current at 0.2 V exceeded a 45 µA target. At a 100 µs reset pulse width, most cycles exhibited abrupt current jumps that exceeded the target current, whereas at a 100 ns width, the programmed current increased gradually in all cycles, enabling precise conductance tuning. Cycle-to-cycle variation decreased by more than 50% as the reset pulse width was reduced, indicating more uniform filament disruption and regrowth. These findings demonstrate that controlling reset pulse width offers a straightforward route to reliable, linear multilevel operation in Al_2_O_3_/TiO_x_-based RRAM.

## 1. Introduction

Resistive random access memory (RRAM) has emerged as a highly promising non-volatile memory (NVM) technology, garnering substantial advantage within the computing-in-memory (CIM) application field owing to its low power consumption, rapid switching speeds, and innate capability for supporting multiple, distinct conductance states [1,2]. Moreover, the architecture of RRAM is remarkably straightforward, consisting of only two terminals in a metal–insulator–metal (MIM) configuration; this inherent simplicity facilitates the realization of high-density cross-point arrays, which are indispensable for the continued scaling of next-generation memory architectures [3]. Among the various resistive switching mechanisms under investigation, filamentary switching—where discrete, conductive filaments form and rupture within the insulating oxide layer—is by far the most widely adopted approach in practical RRAM devices. Common oxide materials employed for this purpose include HfO_2_ [4,5,6], Al_2_O_3_ [7,8], and TiO_2_ [9,10,11], each of which supports filament formation via a localized dielectric breakdown process. Despite these advantageous attributes, RRAM devices are not without their own set of challenges. Critical issues such as current overshoot during set pulses [12], progressive degradation of retention and endurance performance over extended cycling [13,14], and abrupt and uncontrolled switching characteristics [15] continue to hinder device reliability. In particular, the tendency for the set operation to trigger sudden, steep increases in current makes it exceedingly difficult to achieve the finely graded, analog conductance modulation that is a prerequisite for reliable synaptic emulation in CIM hardware.

Recent studies have repeated that using overly high reset pulse amplitudes can unintentionally induce sharp increases in the read current when the subsequent set pulse is applied [16]. Moreover, comparative analyses of the high-resistance state (HRS) have shown that devices with a weaker HRS tend to show a more gradual rise in current during the set operation [17], indicating that the physical and electrical state established during reset has a decisive part in the behavior of the following set. These findings highlight the necessity of developing a carefully optimized reset scheme to achieve more linear, analog-like switching and improve overall device reliability. Until now, prior research has focused primarily on adjusting the amplitude of the reset pulse as the key tuning parameter, leaving other critical pulse attributes—especially its width—largely overlooked. Since the width of a reset pulse dictates how quickly and to what extent the conductive filament undergoes rupture and subsequent reformation, it follows that shortening this duration could dramatically reduce the sudden current surges commonly seen during set operations. In turn, this should promote a more gradual, finely controllable increment of conductance.

Accordingly, in this study, we report on the fabrication and systematic evaluation of Al_2_O_3_/TiO_x_-based RRAM devices subjected to reset pulses of varying widths—specifically 100 µs, 10 µs, 1 µs, and 100 ns. By applying these reset pulses prior to each set sequence and then quantitatively and statistically analyzing the resulting current–voltage responses, we assess the degree to which reset pulse width modulation can be utilized to realize analog-like, gradual conductance tuning. Through this approach, we aim to establish reset pulse width as a novel and effective control key for optimizing the linearity and precision of RRAM synaptic weights.

## 2. Experimental Methods

**Figure 1:**
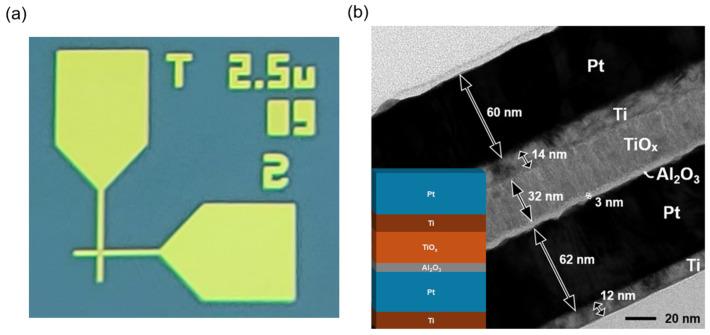
RRAM device fabrication: (**a**) top-view optical microscopy (OM) image of 2.5 µm × 2.5 µm single cell; (**b**) cross-sectional transmission electron microscopy (TEM) image of Ti (12 nm)/Pt (62 nm)/Al_2_O_3_ (3 nm)/TiO_x_ (32 nm)/Ti (14 nm)/Pt (60 nm) stack with a corresponding schematic diagram.

The fabrication process began on a Si wafer coated with 200 nm of SiO_2_. First, a 12 nm titanium adhesion layer and a 62 nm platinum bottom electrode (BE) were sequentially deposited by electron beam evaporation under high-vacuum conditions to ensure uniform film coverage. The bilayer resistive switching stack was then formed: a 3 nm Al_2_O_3_ layer was conformally grown by atomic layer deposition, followed by a 32 nm TiO_x_ layer deposited via DC reactive sputtering from a Ti target, with precise thickness control to optimize filament formation. Finally, a top electrode (TE) stack comprising 14 nm Ti and 60 nm Pt was deposited by e-beam evaporation, completing the symmetric Ti/Pt contacts. Figure 1a shows an optical microscopy (OM) image of the fabricated single-cell device, with the active area defined as 2.5 µm × 2.5 µm. The device stack—Ti (12 nm)/Pt (62 nm)/Al_2_O_3_ (3 nm)/TiO_x_ (32 nm)/Ti (14 nm)/Pt (60 nm)—is clearly observed in the cross-sectional transmission electron microscopy (TEM) image with the stack diagram in Figure 1b, confirming layer integrity and thickness accuracy.

In this work, a 3 nm thick Al_2_O_3_ film was employed as the resistive switching layer. Although this thickness is not necessarily optimized, it was chosen to ensure sufficient insulation characteristics during device operation. Previous studies have identified the Al_2_O_3_ thickness as an important parameter affecting forming voltage and device yield, particularly in crossbar array structures. For example, a thickness of 1.5 nm was reported to provide low forming voltage and high yield [18].

**Figure 2:**
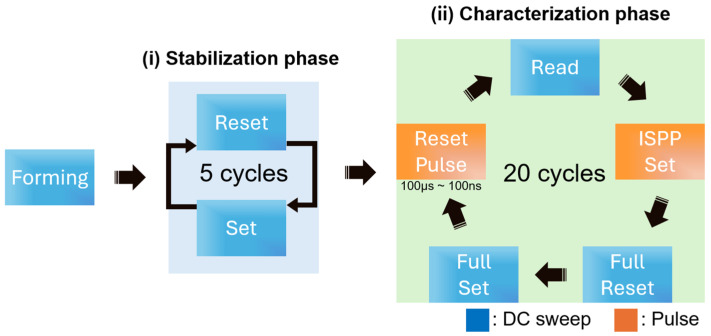
Experiment flow chart to evaluate the effect of reset pulse width on gradual set operation, consisting of two phases after forming: (**i**) stabilization phase, (**ii**) characterization phase.

To identify the influence of reset pulse width on device performance, the measurement protocol was divided into two distinct phases: (i) a stabilization phase and (ii) a characterization phase, as illustrated in Figure 2. The experimental sequence began with a forming process in which a DC voltage sweep was applied up to 4 V under a strict compliance current (I_cc_) limit of 200 µA. This initial forming step serves to initiate and localize conductive filament formation within the Al_2_O_3_/TiO_x_ stack, establishing a well-defined low-resistance path for all subsequent operations.

Upon successful forming, the stabilization phase was executed to ensure reliable, repeatable switching behavior across the array. During this phase, five consecutive DC reset–set cycles were carried out: each reset sweep ruptured the filament, driving the device into its HRS, while each set sweep reformed the filament back into the LRS. By repeatedly rupturing and reforming the filament under controlled compliance current conditions, this stabilization phase progressively minimized cycle-to-cycle variation, thereby creating uniform filaments from which the effects of reset pulse width could be unambiguously assessed. During the stabilization phase, the DC reset was performed at −1.4 V without any compliance current limit, followed by a DC set sweep up to 1.0 V with the compliance current set to 1 mA.

Following the stabilization phase, the characterization phase systematically probed the effect of varying reset pulse widths on set switching behavior. In each of the twenty measurement cycles per condition, a reset pulse with a fixed amplitude of −1.5 V was applied to the top Pt electrode, while the bottom Ti electrode remained grounded. The reset pulse width was varied across four decades—100 µs, 10 µs, 1 µs, and 100 ns—to evaluate how temporal modulation of the reset influences the subsequent set operation. Immediately after each reset, a DC read operation at 0.2 V was performed to confirm that the device had indeed returned to the HRS; only upon verification of a successful reset did the protocol proceed to the set step.

For the set operation, an incremental step pulse programming (ISPP) scheme was employed, in which the set pulse amplitude gradually increased until the read current exceeded the 45 µA target. Once the target read current was reached, the programming sequence was terminated for that cycle. To guarantee consistent initial conditions and to avoid residual filament fragments biasing the next cycle, a full DC reset and a full DC set were performed. In the DC characterization protocol, the reset operation was carried out by sweeping the voltage negatively up to −1.4 V without imposing any I_cc_ limit, allowing the conductive filament to be fully ruptured and the device to reliably reach its high-resistance state. For the subsequent DC set measurement, the applied voltage gradually increased from 0 V to a maximum of 1 V under a strict compliance current of 1 mA. These full resets and full sets were applied between every characterization cycle.

All DC electrical characteristics—forming, set, and reset voltages and on/off resistances—were recorded using a Keithley 4200A-SCS Semiconductor Parameter Analyzer (Keithley Instruments, Cleveland, OH, USA). Static I-V sweeps under controlled I_cc_ conditions provided detailed insight into the device’s quasi-static switching thresholds. In parallel, transient switching behavior, including potentiation and depression dynamics, was captured using a 4225-PMU pulse measurement unit (Keithley Instruments, Cleveland, OH, USA). In pulse mode, programmable trains of voltage pulses (with precisely controlled amplitude, width, and inter-pulse delay) were applied to the Pt top electrode, while the Ti bottom electrode was held on the ground. The 4225-PMU’s sub-nanosecond rise and fall times and high precision timing control enabled accurate recording of transient current responses and the construction of real-time resistance states.

All measurements were conducted at room temperature (25 °C) in ambient atmospheric conditions to reflect standard operating environments. To ensure statistical robustness and reproducibility, each data point—whether from DC I-V sweeps or pulse-mode measurements—represents the average of at least twenty repeated cycles under identical test conditions. This protocol provides a comprehensive and statistically significant dataset for evaluating how reset pulse width modulation can be leveraged to optimize the linearity, precision, and stability of set switching in Al_2_O_3_/TiO_x_-based RRAM devices.

## 3. Results and Discussion

### 3.1. Switching Characteristics of Resistive Random Access Memory

**Figure 3:**
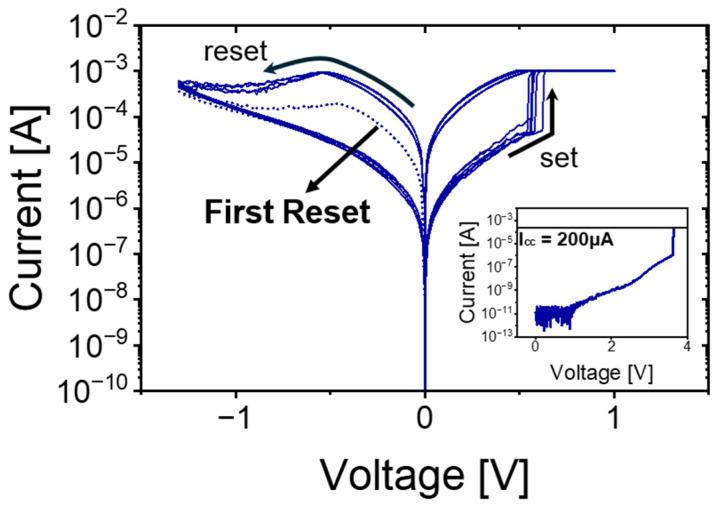
I-V characteristics of set and reset sweeps of the RRAM device for 5 cycles. The right inset shows the forming I-V curve with a compliance current of 200 µA.

The quasi-static I-V response of the Al_2_O_3_/TiO_x_ RRAM cells is depicted in Figure 3, where both set and reset sweeps were recorded under DC conditions, and key features have been highlighted with arrows. The x-axis denotes the applied DC sweep voltage for each resistance state (HRS and LRS), while the y-axis shows the resulting current measured during those dual voltage sweeps. During the very first reset cycle (following forming), the device exhibits a relatively low leakage current throughout the voltage sweep, as indicated by the left-pointing arrow in the plot. This suppressed current level arises because the initial conductive filament, created under a forming I_cc_ of only 200 μA, remains rather narrow and poorly percolated. In contrast, all subsequent set operations were carried out with a substantially higher I_cc_ of 1 mA, which drives the growth of a thicker, more stable filament. As a result, later reset sweeps demonstrate markedly higher reset currents reflecting the increased robustness of the filament structure.

Moreover, the envelope of the reset curves becomes progressively tighter over repeated cycles: the cycle-to-cycle variation in the reset current between the second and fifth sweeps. This reduction in variability signals that the conductive path stabilizes over successive sets/resets. These observations confirm that increasing the I_cc_ during sets strengthens the filament, leading to higher—and more consistent—reset currents, while the initial low-I_cc_ forming produces a fragile filament with correspondingly low reset current. Such behavior underscores the importance of carefully selecting forming and set compliance currents to balance device endurance, variability, and operational margins in Al_2_O_3_/TiO_x_–based RRAM.

**Figure 4:**
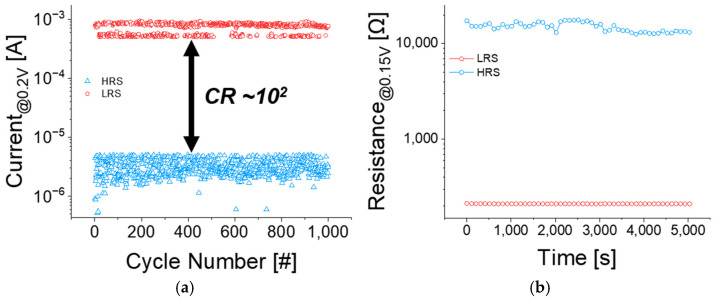
Endurance and retention characteristics: (**a**) LRS/HRS endurance—1000 cycles with current ratio (CR)—10^2^ at read 0.2 V; (**b**) LRS/HRS retention—5000 s at read 0.15 V.

Endurance and retention measurements were conducted, as shown in Figure 4. For endurance evaluation, set (1.4 V~1.6 V, 10 µs), reset (−2.4 V~−1.8 V, 10 µs), and read (0.2 V, 10 µs) pulses were applied repeatedly via the ISPP method, as shown in Figure 4a. The device maintained distinguishable resistance states for over 10^3^ cycles, with a stable current ratio (CR) of approximately 10^2^ between the LRS and HRS. In Figure 4b, additionally, retention characteristics were evaluated for up to 5000 s. Both LRS and HRS were successfully preserved throughout the measurement period, confirming the stability of the programmed resistance states. The degradation of the HRS occurs because atoms diffuse within the conductive filaments (CFs), and these diffused atoms form a percolation path at the ruptured sites. While endurance and retention measurements confirm the uniformity of the Al_2_O_3_/TiO_x_-based RRAM, the stochastic formation and rupture of conductive filaments in the pristine state require minimal device-to-device variation to ensure reliability [19].

**Figure 5:**
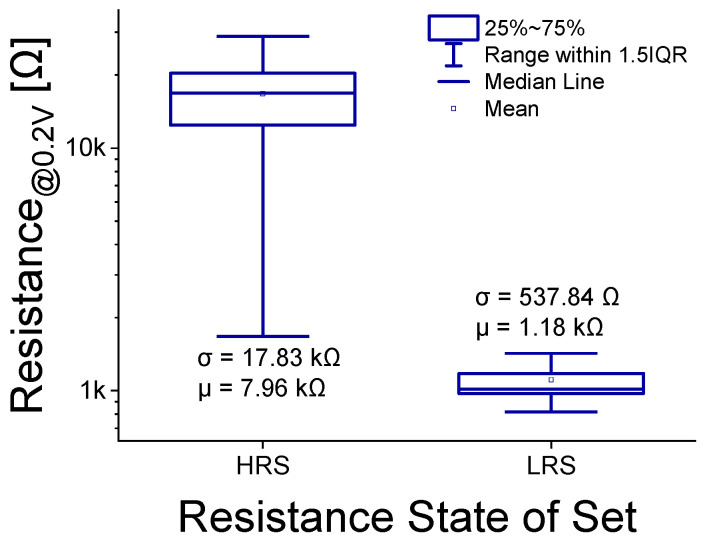
LRS and HRS variation box plots at read 0.2 V from 30 single-cell devices.

To evaluate device variation, LRS and HRS resistances were measured for 30 devices with an area of 2.5 µm × 2.5 µm, as shown in Figure 5. After electroforming each device in the pristine state, reset and set cycles were performed sequentially using a DC voltage sweep. During the dual-sweep set process, HRS and LRS currents were read at 0.2 V. As shown in Figure 5, device-to-device variation was more pronounced in the HRS region. LRS variation is governed by the number and size of conductive filaments (CFs), whereas HRS variation depends on the length of the ruptured CFs (i.e., the tunneling gap). Because the tunneling current depends exponentially on the gap length, HRS variation exceeds LRS variation [3,20].

### 3.2. Characterization of ISPP Set Behavior Depending on Reset Pulse Width

**Figure 6:**
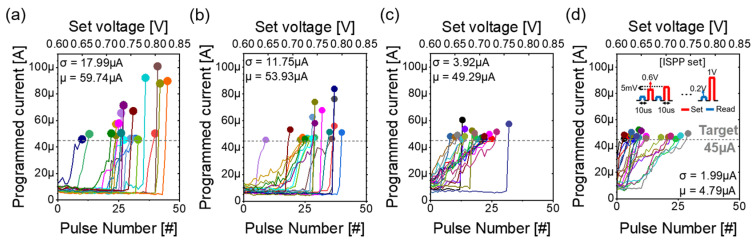
Incremental step programming pulse (ISPP) set characteristics over 20 cycles with distinguished colors depending on the different reset pulse widths: (**a**) 100 µs, (**b**) 10 µs, (**c**) 1 µs, and (**d**) 100 ns.

Figure 6 presents the ISPP set characteristics for Al_2_O_3_/TiO_x_ RRAM devices that were preconditioned with four distinct reset pulse widths: (a) 100 µs, (b) 10 µs, (c) 1 µs, and (d) 100 ns. The lower x-axis indicates the pulse number, the upper x-axis shows the set pulse amplitude, and the y-axis represents the magnitude of the programmed current, as shown in Figure 6a–d. In each of these measurement cycles, the set pulse amplitude was incrementally ramped from 0.6 V to 1.0 V in precise 5 mV steps, and the resulting read current was recorded under a constant 0.2 V read bias. The programming algorithm was configured with a target current of 45 µA, and as soon as the read current surpassed this target threshold, the programming sequence for that cycle was immediately terminated.

In Figure 6a, corresponding to the longest reset pulse width of 100 µs, the read current trace remains almost flat and unchanged throughout the lower portion of the voltage sweep, before exhibiting a pronounced, abrupt increase at a critical set voltage—this jump indicates a sudden and uncontrolled conductive filament formation. By contrast, Figure 6d, which depicts behavior under the shortest reset pulse width of 100 ns, reveals a markedly different profile: as the set voltage increases, the read current rises in a smooth, continuous fashion, without sudden jumps. This more gradual current ramp clearly reflects enhanced control over the conductance growth process, allowing each incremental voltage step to contribute proportionally to filament thickness.

Moreover, when comparing Figure 6a through Figure 6d, it becomes evident that the cycle-to-cycle variation in programmed current systematically decreases as the reset pulse width is shortened. Quantitatively, we observe that the standard deviation of the final programmed current across the 20 cycles drops from approximately about 18 µA at 100 μs down to less than about 4.8 µA at 100 ns, demonstrating a threefold reduction in variability. This trend underscores the principle that more gradual current increases are intrinsically associated with lower dispersion in the final conductance values.

This tendency suggests that shorter reset pulses enable more precise control over filament growth during the subsequent ISPP operation. The reduced variation is likely due to less extensive filament disruption during shorter reset pulses, which preserves more of the filament’s residual structure and yields a more controllable and gradual conductance increase during the set process [21]. Overall, the data confirm that employing shorter reset pulse widths contributes significantly to achieving gradual conductance changes with the ISPP scheme, thereby markedly improving the linearity, precision, and reproducibility of the set process in Al_2_O_3_/TiO_x_ RRAM devices.

**Figure 7:**
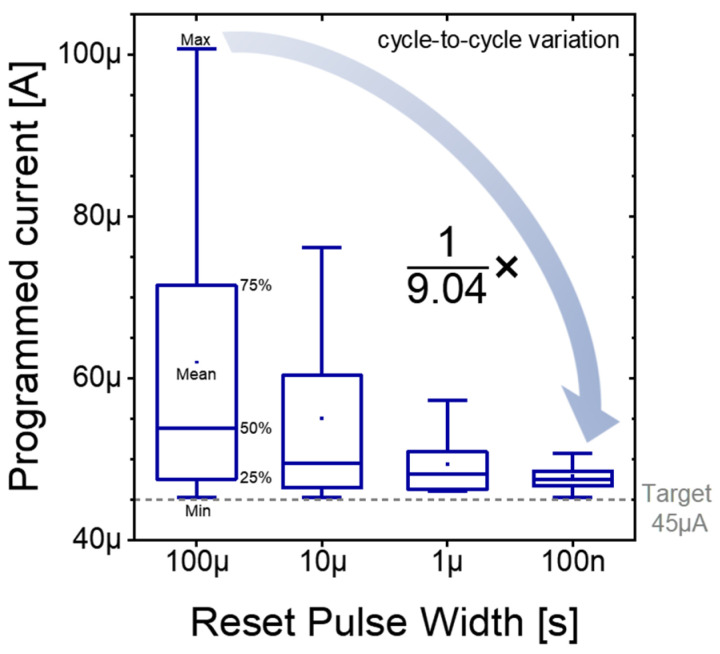
Cycle-to-cycle variation in the final programmed current over 20 cycles for reset pulse widths ranging from 10^−4^ s to 10^−7^ s.

To compare the variation in read current when it first exceeds the target value of 45 µA, Figure 7 presents box plots that clearly illustrate how the distribution contracts as the reset pulse widths are progressively shortened. The x-axis corresponds to the four reset pulse width conditions, and the y-axis displays the distribution of the programmed current for each condition. Under the longest 100 µs condition, the programming currents are highly scattered: the numerous cycles overshoot this target by large margins, often exceeding 60 µA. This broad and erratic spread reflects the inability of long reset pulses to consistently prepare the device for a controlled set event—excessive filament dissolution during the lengthy reset leads to unpredictable regrowth dynamics, resulting in abrupt current jumps when the set pulse is applied.

When the reset pulse width is reduced to 10 µs, the spread of read currents begins to tighten noticeably. Fewer cycles exhibit extreme overshoots, and the interquartile range narrows, indicating that partial filament rupture under shorter pulses leaves behind more stable residual filaments that guide a more uniform regrowth during sets. A further reduction to 1 µs continues this trend: the median programmed current shifts significantly toward the target 45 µA, and the number of outlier cycles drops dramatically. Even at this intermediate pulse width, one observes a marked improvement in programming predictability, as the device transitions from the high-resistance to the low-resistance state in a more stepwise, controlled fashion.

At the shortest 100 ns width, almost all programming cycles terminate extremely close to the target current. Here, both the median and the upper and lower quartiles align near 45 µA, and only rare, minor deviations appear beyond this range. These observations demonstrate that shorter reset pulse widths yield a progressively more gradual rise in programmed current, enabling the programming sequence to halt reliably at or near the desired threshold. The smoother current slope seen with 100 ns pulses underscores how minimal filament disruption fosters more deterministic regrowth: only the weakest links in the filament network are broken, preserving a scaffold that guides subsequent set pulses.

Consequently, such a gradual current slope not only enhances programming accuracy but also significantly improves the repeatability of the resulting resistance state. The tight clustering of programmed currents under short pulse conditions translates to lower cycle-to-cycle variability—a critical requirement for analog memory and neuromorphic applications demanding deterministic weight updates. By ensuring that each weight update lands predictably at the intended conductance level, reset pulse width optimization emerges as a powerful strategy for achieving high-precision, low-variance programming in next-generation RRAM-based CIM systems.

### 3.3. NN Simulation Including Resistance Variations

Figure 7 presents the current values at which the target current was first exceeded for each cycle, across all reset pulse widths. This figure clearly shows that as the reset pulse width decreases, the variation in the programming current becomes smaller. To further investigate the impact of this variation on programming accuracy, we conducted a simple neural network (NN) simulation. The neural network consisted of three fully connected layers (784-256-128-10). The MNIST dataset was used for this experiment, with a learning rate of 0.0005 and 50 training epochs. Each training run was repeated 20 times to evaluate consistency in accuracy. Using the measured variation from the RRAM device, we applied it to all weights of the neural network model to simulate its effect.

**Figure 8:**
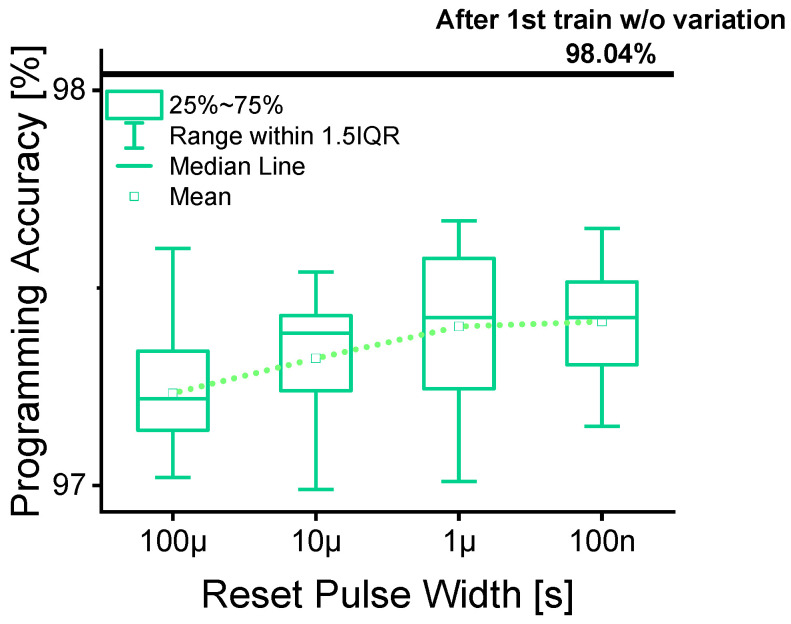
Programming accuracy box plot relying on cycle-to-cycle variation in the last programmed current over 20 cycles for reset pulse widths.

As shown in Figure 8, the results indicated that as the reset pulse width decreased, the programming accuracy of the neural network improved. However, since the simulation included real-world variation data, we observed that the overall accuracy after training was slightly lower compared to an ideal case without variation. This emphasizes the importance of minimizing device variation to maintain high programming accuracy in neuromorphic computing applications.

### 3.4. Switching Mechanisms of Resistive Random Access Memory

**Figure 9:**
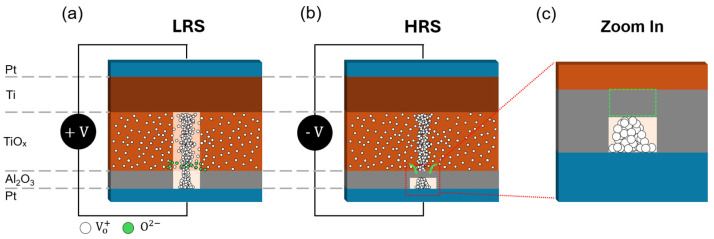
Schematic illustration of the switching mechanism in Al_2_O_3_/TiO_x_-based RRAM: (**a**) low-resistance state (LRS); (**b**) high-resistance state (HRS); (**c**) enlarged view of the Al_2_O_3_ layer in (**b**), highlighting structural reconfiguration under the HRS condition and the green box shows reoxidation in Al_2_O_3_ layer.

Figure 9 depicts a schematic cross-section of the active Al_2_O_3_/TiO_x_ stack—rather than the full device architecture—highlighting the core layers responsible for resistive switching. In this simplified representation, a Ti/Pt TE stack interfaces with a 3 nm Al_2_O_3_ layer, which in turn overlies a 32 nm TiO_x_ layer before reaching the Ti/Pt BE stack. Under a positive bias applied to the TE (with the BE grounded), the Al_2_O_3_/TiO_x_ interface induces ionization of lattice oxygen. O^2−^ ions migrate into the TiO_x_ layer, leaving behind oxygen vacancies that coalesce into a continuous conductive filament spanning the bilayer, as shown in Figure 9a, thereby driving the device into the low-resistance state (LRS). During this process, partial ionization in the Al_2_O_3_ also contributes additional O^2−^ ions drifting toward the TiO_x_, further stabilizing the filament network.

When the polarity is reversed—i.e., a negative bias at the TE with the BE grounded—the local electric field drives O^2−^ ions back toward the Al_2_O_3_ layer. These ions recombine with Al^3+^ cations in the near-electrode region of the bilayer, initiating an oxidative “healing” of the Al_2_O_3_ layer, as shown in Figure 9b. This reoxidation undermines the weakest segments of the filament, causing it to thin and eventually rupture. Once the filament disconnects, electronic conduction is suppressed, and the device returns to the HRS. A high-resolution inset in Figure 9c zooms into the Al_2_O_3_ layer during HRS, revealing how O^2−^ ions refill oxygen-vacant lattice sites, restoring Al_2_O_3_. This reversible cycle of vacancy generation, vacancy migration, and oxide reformation within the Al_2_O_3_/TiO_x_ stack is the fundamental process enabling reliable, repeatable LRS and HRS switching in these RRAM cells.

### 3.5. Switching Mechanisms of AC Resistive Random Access Memory

**Figure 10:**
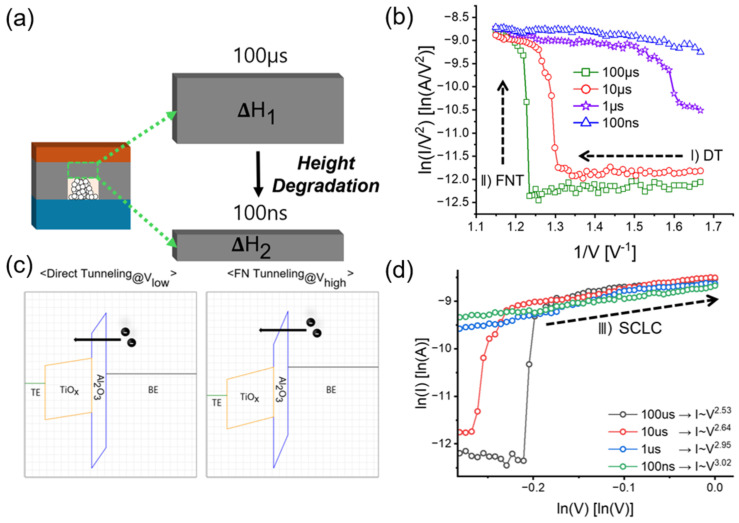
Schematic illustration of the switching mechanism in Al_2_O_3_/TiO_x_-based RRAM: (**a**) difference of Al_2_O_3_ barrier layer height depending on reset pulse width; (**b**) for 100 µs and 10 µs pulses, direct tunneling (DT) is evident at low voltages and Fowler–Nordheim tunneling (FNT) at high voltages; the 1 µs curve still displays both DT and FNT contributions, whereas in the 100 ns case, tunneling signatures are nearly absent; (**c**) energy band diagrams illustrating the switching mechanisms: at low applied voltages, electron transport occurs via DT through the Al_2_O_3_ barrier, while at higher voltages FNT under a thinned triangular barrier dominates; (**d**) above the FNT onset voltage, the slope drops sharply, consistent with space-charge-limited conduction (SCLC) following Child’s law.

Al_2_O_3_ serves as a diffusion limiting layer (DLL) due to its high activation energy and low oxygen diffusion coefficient [22]. As shown in Figure 10a, increasing the reset pulse width yields a higher resistance state [23], implying that the Al_2_O_3_ barrier layer becomes thicker with longer pulse durations. A thicker Al_2_O_3_ layer (e.g., 1.5–1.8 nm) requires a higher voltage for forming or set operations to induce breakdown [18]. Although the pristine Al_2_O_3_ layer is initially 3 nm thick, even after a reset pulse is applied, longer pulse widths necessitate a greater number of ISPP set pulses for successful programming.

Figure 10b shows that longer reset pulse widths lead to a more pronounced increase in current at higher voltages. This sudden current increase can be explained by the combined mechanisms of direct tunneling (DT) and Fowler–Nordheim tunneling (FNT). During the ISPP set process, electrons tunnel DLL via DT at low voltages into the TiO_x_ layer, as shown in Figure 10c. As the voltage increases, bond breakage progresses and electric fields intensify, reducing the triangular barrier thickness and allowing electrons to tunnel via FNT into the TiO_x_ layer [24,25,26]. Accordingly, as seen in Figure 10b, the negative slope at low voltages is attributed to DT, while an increase in reset pulse width results in a larger number of required pulses and a stronger electric field, leading to a near-vertical positive slope. However, in the case of the 1 µs reset pulse, the current increase begins at a lower voltage compared to the 10 µs and 100 µs cases, indicating that DT and FNT both still play a role. Interestingly, after reset pulses of 100 µs to 1 µs, the slope of the I-V curve becomes more gradual, resembling the behavior seen at 100 ns. This suggests that the oxide barrier (ΔH) becomes too thin to support sufficient FNT, leading to a different mechanism [27].

This behavior can be explained by the space charge limited conduction (SCLC) mechanism [16]. In SCLC, current exhibits an abrupt increase at a specific voltage (the trap-filled limit voltage), followed by a quadratic dependence on voltage, consistent with Child’s law (I∼V^2^) [28]. When related to the FNT mechanism previously discussed, electrons tunnel through the Al_2_O_3_ barrier and fill the V_O_^2+^ traps in the TiO_x_ layer. Once these traps are fully occupied, the current begins to increase in accordance with the I∼V^2^; relationship. Figure 10d shows the ln I–ln V curves during ISPP set operations after reset pulses of various widths (100 µs to 100 ns). The direction behavior consists of Child’s law. Although the fitting was performed based on the 100 µs case, the curves for 1 µs and 100 ns also exhibit a similar slope from relatively lower voltages. The current–voltage relationships follow the power laws of I~V^2.53^, I~V^2.64^, I~V^2.95^, and I~V^3.02^ for pulse widths of 100 µs, 10 µs, 1 µs, and 100 ns, respectively.

Finally, to assume the significance of the case with a 100 ns reset pulse. During the subsequent ISPP set process, the contribution from FNT is relatively weak because most traps are already filled with electrons. Thus, the conduction mechanism is better explained by the trap-free SCLC (TF-SCLC) model.

## 4. Conclusions

This study investigated the effect of reset pulse width on the switching behavior of Al_2_O_3_/TiO_x_-based RRAM devices, with the aim of suppressing abrupt increases in set currents and achieving more gradual switching characteristics. To this end, the device was fabricated, and two measurement phases were employed: (i) a stabilization phase to ensure device stability, and (ii) a characterization phase focusing on device behavior in the HRS. As a result, the reset pulse width of 100 µs led to abrupt increases in the programmed current in most cycles, often significantly overshooting the target current. In contrast, at a reset pulse width of 100 ns, the programmed current gradually increased across all cycles, enabling more precise programming near the target current level. Furthermore, it was observed that the cycle-to-cycle variation in programmed current decreased as the reset pulse width was reduced from 100 µs to 100 ns. These results demonstrate that reducing the reset pulse width is critical for achieving precise tuning of the conductance state in Al_2_O_3_/TiO_x_-based RRAM devices. Such control over switching dynamics not only improves device reliability and endurance but also enhances the linearity required for neuromorphic computing and high-density storage applications, paving the way for advanced CIM systems.

## Data Availability

The raw data supporting the conclusions of this article will be made available by the authors on request.
